# Supplementary material for: Pyruvate Dehydrogenase Kinase 1 inhibition mediated oxidative phosphorylation enhancement in cartilage promotes osteoarthritis progression
Source: BMC Musculoskelet Disord. 2023 Jul 20;24:597. doi: 10.1186/s12891-023-06585-6 (PMC10357736; doi:10.1186/s12891-023-06585-6)
Supplement: Supplementary file 1 — Additional file 1. [file 12891_2023_6585_MOESM1_ESM.zip › BP.Gsea.1653623667859/GOBP_MITOCHONDRIAL_GENE_EXPRESSION.html]

Details for gene set GOBP\_MITOCHONDRIAL\_GENE\_EXPRESSION[GSEA]

|  || Dataset | 55457\_collapsed\_to\_symbols.cls.cls #OA\_versus\_normal.cls.cls #OA\_versus\_normal\_repos |
| Phenotype | cls.cls#OA\_versus\_normal\_repos |
| Upregulated in class | OA |
| GeneSet | GOBP\_MITOCHONDRIAL\_GENE\_EXPRESSION |
| Enrichment Score (ES) | 0.36605006 |
| Normalized Enrichment Score (NES) | 1.631951 |
| Nominal p-value | 0.0056179776 |
| FDR q-value | 0.2202472 |
| FWER p-Value | 1.0 |
Table: GSEA Results Summary

  

Fig 1: Enrichment plot: GOBP\_MITOCHONDRIAL\_GENE\_EXPRESSION      
 Profile of the Running ES Score & Positions of GeneSet Members on the Rank Ordered List

  

| SYMBOL | TITLE | RANK IN GENE LIST | RANK METRIC SCORE | RUNNING ES | CORE ENRICHMENT || 1 | TRMT5 | tRNA methyltransferase 5 [Source:HGNC Symbol;Acc:HGNC:23141] | 125 | 0.879 | 0.0259 | Yes |
| 2 | MRPL16 | mitochondrial ribosomal protein L16 [Source:HGNC Symbol;Acc:HGNC:14476] | 132 | 0.874 | 0.0608 | Yes |
| 3 | TUFM | "Tu translation elongation factor, mitochondrial [Source:HGNC Symbol;Acc:HGNC:12420]" | 189 | 0.828 | 0.0901 | Yes |
| 4 | MRPS14 | mitochondrial ribosomal protein S14 [Source:HGNC Symbol;Acc:HGNC:14049] | 207 | 0.814 | 0.1217 | Yes |
| 5 | MRPL57 | mitochondrial ribosomal protein L57 [Source:HGNC Symbol;Acc:HGNC:14514] | 314 | 0.740 | 0.1435 | Yes |
| 6 | COA3 | cytochrome c oxidase assembly factor 3 [Source:HGNC Symbol;Acc:HGNC:24990] | 597 | 0.625 | 0.1468 | Yes |
| 7 | NGRN | "neugrin, neurite outgrowth associated [Source:HGNC Symbol;Acc:HGNC:18077]" | 741 | 0.583 | 0.1593 | Yes |
| 8 | TACO1 | translational activator of cytochrome c oxidase I [Source:HGNC Symbol;Acc:HGNC:24316] | 923 | 0.536 | 0.1669 | Yes |
| 9 | MRPS16 | mitochondrial ribosomal protein S16 [Source:HGNC Symbol;Acc:HGNC:14048] | 994 | 0.519 | 0.1825 | Yes |
| 10 | PRORP | protein only RNase P catalytic subunit [Source:HGNC Symbol;Acc:HGNC:19958] | 1013 | 0.515 | 0.2020 | Yes |
| 11 | MTIF2 | mitochondrial translational initiation factor 2 [Source:HGNC Symbol;Acc:HGNC:7441] | 1043 | 0.508 | 0.2203 | Yes |
| 12 | NDUFA7 | NADH:ubiquinone oxidoreductase subunit A7 [Source:HGNC Symbol;Acc:HGNC:7691] | 1097 | 0.496 | 0.2363 | Yes |
| 13 | TSFM | "Ts translation elongation factor, mitochondrial [Source:HGNC Symbol;Acc:HGNC:12367]" | 1120 | 0.492 | 0.2546 | Yes |
| 14 | QRSL1 | glutaminyl-tRNA amidotransferase subunit QRSL1 [Source:HGNC Symbol;Acc:HGNC:21020] | 1250 | 0.467 | 0.2635 | Yes |
| 15 | MRPL58 | mitochondrial ribosomal protein L58 [Source:HGNC Symbol;Acc:HGNC:5359] | 1298 | 0.459 | 0.2784 | Yes |
| 16 | MRPS11 | mitochondrial ribosomal protein S11 [Source:HGNC Symbol;Acc:HGNC:14050] | 1305 | 0.457 | 0.2965 | Yes |
| 17 | SHMT2 | serine hydroxymethyltransferase 2 [Source:HGNC Symbol;Acc:HGNC:10852] | 1518 | 0.426 | 0.2972 | Yes |
| 18 | RCC1L | RCC1 like [Source:HGNC Symbol;Acc:HGNC:14948] | 1559 | 0.419 | 0.3111 | Yes |
| 19 | MRPS7 | mitochondrial ribosomal protein S7 [Source:HGNC Symbol;Acc:HGNC:14499] | 1650 | 0.406 | 0.3205 | Yes |
| 20 | IARS2 | "isoleucyl-tRNA synthetase 2, mitochondrial [Source:HGNC Symbol;Acc:HGNC:29685]" | 1685 | 0.401 | 0.3342 | Yes |
| 21 | FASTK | Fas activated serine/threonine kinase [Source:HGNC Symbol;Acc:HGNC:24676] | 1834 | 0.380 | 0.3380 | Yes |
| 22 | MRPS28 | mitochondrial ribosomal protein S28 [Source:HGNC Symbol;Acc:HGNC:14513] | 1865 | 0.377 | 0.3510 | Yes |
| 23 | MTO1 | mitochondrial tRNA translation optimization 1 [Source:HGNC Symbol;Acc:HGNC:19261] | 2181 | 0.340 | 0.3402 | Yes |
| 24 | MRPL23 | mitochondrial ribosomal protein L23 [Source:HGNC Symbol;Acc:HGNC:10322] | 2210 | 0.337 | 0.3517 | Yes |
| 25 | MRPS18A | mitochondrial ribosomal protein S18A [Source:HGNC Symbol;Acc:HGNC:14515] | 2295 | 0.327 | 0.3584 | Yes |
| 26 | FASTKD3 | FAST kinase domains 3 [Source:HGNC Symbol;Acc:HGNC:28758] | 2473 | 0.306 | 0.3570 | Yes |
| 27 | GATB | glutamyl-tRNA amidotransferase subunit B [Source:HGNC Symbol;Acc:HGNC:8849] | 2515 | 0.302 | 0.3661 | Yes |
| 28 | WARS2 | "tryptophanyl tRNA synthetase 2, mitochondrial [Source:HGNC Symbol;Acc:HGNC:12730]" | 3014 | 0.248 | 0.3373 | No |
| 29 | C1QBP | complement C1q binding protein [Source:HGNC Symbol;Acc:HGNC:1243] | 3083 | 0.241 | 0.3418 | No |
| 30 | MRPL44 | mitochondrial ribosomal protein L44 [Source:HGNC Symbol;Acc:HGNC:16650] | 3306 | 0.220 | 0.3334 | No |
| 31 | SARS2 | "seryl-tRNA synthetase 2, mitochondrial [Source:HGNC Symbol;Acc:HGNC:17697]" | 3411 | 0.210 | 0.3338 | No |
| 32 | MRPS15 | mitochondrial ribosomal protein S15 [Source:HGNC Symbol;Acc:HGNC:14504] | 3475 | 0.205 | 0.3372 | No |
| 33 | NSUN3 | NOP2/Sun RNA methyltransferase 3 [Source:HGNC Symbol;Acc:HGNC:26208] | 3612 | 0.192 | 0.3344 | No |
| 34 | GFM1 | G elongation factor mitochondrial 1 [Source:HGNC Symbol;Acc:HGNC:13780] | 4011 | 0.155 | 0.3097 | No |
| 35 | HSD17B10 | hydroxysteroid 17-beta dehydrogenase 10 [Source:HGNC Symbol;Acc:HGNC:4800] | 4060 | 0.150 | 0.3120 | No |
| 36 | MRPS27 | mitochondrial ribosomal protein S27 [Source:HGNC Symbol;Acc:HGNC:14512] | 4088 | 0.147 | 0.3159 | No |
| 37 | TEFM | "transcription elongation factor, mitochondrial [Source:HGNC Symbol;Acc:HGNC:26223]" | 4447 | 0.117 | 0.2927 | No |
| 38 | DARS2 | "aspartyl-tRNA synthetase 2, mitochondrial [Source:HGNC Symbol;Acc:HGNC:25538]" | 4480 | 0.115 | 0.2949 | No |
| 39 | ELAC2 | elaC ribonuclease Z 2 [Source:HGNC Symbol;Acc:HGNC:14198] | 4532 | 0.110 | 0.2954 | No |
| 40 | ALKBH1 | "alkB homolog 1, histone H2A dioxygenase [Source:HGNC Symbol;Acc:HGNC:17911]" | 4765 | 0.090 | 0.2809 | No |
| 41 | MRPS17 | mitochondrial ribosomal protein S17 [Source:HGNC Symbol;Acc:HGNC:14047] | 4898 | 0.080 | 0.2739 | No |
| 42 | RMND1 | required for meiotic nuclear division 1 homolog [Source:HGNC Symbol;Acc:HGNC:21176] | 5084 | 0.066 | 0.2621 | No |
| 43 | HARS1 | histidyl-tRNA synthetase 1 [Source:HGNC Symbol;Acc:HGNC:4816] | 5196 | 0.057 | 0.2557 | No |
| 44 | MRPL12 | mitochondrial ribosomal protein L12 [Source:HGNC Symbol;Acc:HGNC:10378] | 5396 | 0.041 | 0.2419 | No |
| 45 | TARS2 | "threonyl-tRNA synthetase 2, mitochondrial [Source:HGNC Symbol;Acc:HGNC:30740]" | 5576 | 0.026 | 0.2290 | No |
| 46 | MTG1 | mitochondrial ribosome associated GTPase 1 [Source:HGNC Symbol;Acc:HGNC:32159] | 5588 | 0.025 | 0.2291 | No |
| 47 | TFB1M | "transcription factor B1, mitochondrial [Source:HGNC Symbol;Acc:HGNC:17037]" | 5789 | 0.009 | 0.2139 | No |
| 48 | FASTKD5 | FAST kinase domains 5 [Source:HGNC Symbol;Acc:HGNC:25790] | 5982 | -0.003 | 0.1990 | No |
| 49 | PPARGC1B | PPARG coactivator 1 beta [Source:HGNC Symbol;Acc:HGNC:30022] | 6184 | -0.019 | 0.1841 | No |
| 50 | TRMT61B | tRNA methyltransferase 61B [Source:HGNC Symbol;Acc:HGNC:26070] | 6223 | -0.022 | 0.1821 | No |
| 51 | MTERF4 | mitochondrial transcription termination factor 4 [Source:HGNC Symbol;Acc:HGNC:28785] | 6972 | -0.078 | 0.1269 | No |
| 52 | LARS2 | "leucyl-tRNA synthetase 2, mitochondrial [Source:HGNC Symbol;Acc:HGNC:17095]" | 7017 | -0.081 | 0.1268 | No |
| 53 | GARS1 | glycyl-tRNA synthetase 1 [Source:HGNC Symbol;Acc:HGNC:4162] | 7069 | -0.085 | 0.1263 | No |
| 54 | TFB2M | "transcription factor B2, mitochondrial [Source:HGNC Symbol;Acc:HGNC:18559]" | 7112 | -0.089 | 0.1266 | No |
| 55 | MRPS12 | mitochondrial ribosomal protein S12 [Source:HGNC Symbol;Acc:HGNC:10380] | 7328 | -0.104 | 0.1141 | No |
| 56 | FASTKD2 | FAST kinase domains 2 [Source:HGNC Symbol;Acc:HGNC:29160] | 7730 | -0.136 | 0.0883 | No |
| 57 | GATC | glutamyl-tRNA amidotransferase subunit C [Source:HGNC Symbol;Acc:HGNC:25068] | 7852 | -0.144 | 0.0848 | No |
| 58 | MRPS2 | mitochondrial ribosomal protein S2 [Source:HGNC Symbol;Acc:HGNC:14495] | 7863 | -0.146 | 0.0899 | No |
| 59 | MRPS18B | mitochondrial ribosomal protein S18B [Source:HGNC Symbol;Acc:HGNC:14516] | 8145 | -0.166 | 0.0747 | No |
| 60 | LRPPRC | leucine rich pentatricopeptide repeat containing [Source:HGNC Symbol;Acc:HGNC:15714] | 8249 | -0.175 | 0.0738 | No |
| 61 | PTCD3 | pentatricopeptide repeat domain 3 [Source:HGNC Symbol;Acc:HGNC:24717] | 8287 | -0.178 | 0.0781 | No |
| 62 | FASTKD1 | FAST kinase domains 1 [Source:HGNC Symbol;Acc:HGNC:26150] | 8942 | -0.227 | 0.0363 | No |
| 63 | TBRG4 | transforming growth factor beta regulator 4 [Source:HGNC Symbol;Acc:HGNC:17443] | 9818 | -0.302 | -0.0197 | No |
| 64 | YARS2 | tyrosyl-tRNA synthetase 2 [Source:HGNC Symbol;Acc:HGNC:24249] | 10299 | -0.350 | -0.0429 | No |
| 65 | CDK5RAP1 | CDK5 regulatory subunit associated protein 1 [Source:HGNC Symbol;Acc:HGNC:15880] | 10870 | -0.413 | -0.0706 | No |
| 66 | FOXO3 | forkhead box O3 [Source:HGNC Symbol;Acc:HGNC:3821] | 11305 | -0.472 | -0.0853 | No |
| 67 | PRKAA1 | protein kinase AMP-activated catalytic subunit alpha 1 [Source:HGNC Symbol;Acc:HGNC:9376] | 11499 | -0.501 | -0.0800 | No |
| 68 | MTRF1 | mitochondrial translation release factor 1 [Source:HGNC Symbol;Acc:HGNC:7469] | 11517 | -0.503 | -0.0610 | No |
| 69 | POLRMT | RNA polymerase mitochondrial [Source:HGNC Symbol;Acc:HGNC:9200] | 11525 | -0.505 | -0.0410 | No |
| 70 | TRIT1 | tRNA isopentenyltransferase 1 [Source:HGNC Symbol;Acc:HGNC:20286] | 11658 | -0.526 | -0.0300 | No |
| 71 | METTL4 | "methyltransferase 4, N6-adenosine [Source:HGNC Symbol;Acc:HGNC:24726]" | 11976 | -0.589 | -0.0308 | No |
| 72 | TWNK | twinkle mtDNA helicase [Source:HGNC Symbol;Acc:HGNC:1160] | 12069 | -0.612 | -0.0132 | No |
| 73 | SUPV3L1 | Suv3 like RNA helicase [Source:HGNC Symbol;Acc:HGNC:11471] | 12087 | -0.616 | 0.0105 | No |
| 74 | TFAM | "transcription factor A, mitochondrial [Source:HGNC Symbol;Acc:HGNC:11741]" | 12175 | -0.637 | 0.0295 | No |
| 75 | PUS1 | pseudouridine synthase 1 [Source:HGNC Symbol;Acc:HGNC:15508] | 12260 | -0.664 | 0.0499 | No |
Table: GSEA details [plain text format]

  

Fig 2: GOBP\_MITOCHONDRIAL\_GENE\_EXPRESSION      
 Blue-Pink O' Gram in the Space of the Analyzed GeneSet

  

Fig 3: GOBP\_MITOCHONDRIAL\_GENE\_EXPRESSION: Random ES distribution      
 Gene set null distribution of ES for **GOBP\_MITOCHONDRIAL\_GENE\_EXPRESSION**
